# Clinical and Biochemical Manifestations of Depression: Relation to the Neurobiology of Stress

**DOI:** 10.1155/2015/581976

**Published:** 2015-03-24

**Authors:** Phillip W. Gold, Rodrigo Machado-Vieira, Maria G. Pavlatou

**Affiliations:** ^1^National Institute of Mental Health Intramural Research Program, National Institutes of Health, Bethesda, MD 20892, USA; ^2^Unit on Molecular Hormone Action, Program in Reproductive and Adult Endocrinology, Eunice Kennedy Shriver National Institute of Child Health and Human Development, National Institutes of Health, Bethesda, MD 20892, USA

## Abstract

Major depressive disorder (MDD) is a chronic, recurrent, and severe psychiatric disorder with high mortality and medical comorbidities. Stress-related pathways have been directly involved in the pathophysiology and treatment of MDD. The present paper provides an overview on the stress system as a model to understand key pathophysiological paradigms in MDD. These mechanisms involve behavioral, cognitive, and systemic manifestations and are also associated with the mechanisms of action of effective antidepressants. Aspects such as depression subtypes, inflammation, insulin resistance, oxidative stress, and prothrombotic states in critical brain circuits and periphery are critically appraised. Finally, new strategies for approaching treatment-resistant major depression and potential adverse effects associated with this complex and intricate network are highlighted. The authors used PubMed as the database for this review. Each author extracted relevant data and assessed the methodological quality of each study.

## 1. Introduction

Most animal models of depressive behavior depend upon chronic, often inescapable stress paradigms. The CNS changes that accompany these procedures are similar to those seen in humans during neuroimaging and postmortem studies, and they are consistently reversible by multiple classes of antidepressants. This paper describes a CNS stress system that responds to normal or severe stressors in an adaptive way that is often essential for survival. It is this precise system that becomes dysregulated in patients with depression. Thus, depression represents a dysregulation of a normal adaptive system, the stress system. Depressive illness can thus be analogized to another critical adaptive system that becomes dysregulated in the autoimmune disease. Further delineation of the pathophysiology of depression can potentially identify components of a broader stress system than we can currently recognize. In addition, further characterization of the stress system can provide potential targets for new treatments for depressive illness.

Major depression is a heritable disorder that affects approximately 8% of men and 15% of women in the course of their lifetime [1, 2]. For over 75% of patients, major depression is a recurrent illness, characterized by repeated remissions and exacerbations [3]. Over 50% of patients who recover from a first depressive episode will have a second within six months unless they are given maintenance antidepressant treatment [3]. For those who never receive treatment, as many as 15% will succumb to suicide [4–6].

Depression not only causes great mental anguish but also intrudes upon fundamental biological processes that regulate inflammation, coagulation, metabolism, autonomic function, neuroendocrine regulation, sleep, and appetite (reviewed in [5, 7–9]). These disturbances are likely to contribute to the premature coronary artery disease premature osteoporosis and the doubling of mortality in patients with major depression at any age independent of suicide, smoking, or significant physical illness [10–14]. In addition, premenopausal women with major depression have premature osteoporosis and osteopenia [15]. Taking into account the natural history, mental suffering, and medical morbidity associated with major depression, the World Health Organization ranked this disorder as the fourth leading cause of disability worldwide (reviewed in [4, 16]).

Major depression and the stress response share many mediators, circuitries, and phenomenologies. Stress precipitates major depression [17] and influences its severity, duration, and natural history [3, 4, 18, 19]. Depressive illness, like stress system activation, shares a relatively unshifting effect, a shift from complex modes of thought to those that are relatively well-rehearsed or reflexive, and a dysregulation of fundamental biological processes that regulate sleep, appetite, growth, reproduction, and autonomic function [20, 21]. Moreover, the inflammation, metabolic alterations, and the prothrombotic state that characterized major depression also occur during the acute stress response.

This paper will provide an overview of the organization of the stress system as a template for understanding key pathophysiological mechanisms in major depression. These mechanisms are involved not only in the behavioral, cognitive, and systemic manifestations of major depression, but also in the mechanisms of actions of some effective antidepressants. We will also provide a brief overview of strategies for approaching treatment-resistant major depression and a brief review of the major side effects of the principal categories of antidepressant agents.

## 2. MDD Subgroups: Melancholic and Atypical Depression

Major depression is not likely to be a single disorder and has many differing phenotypic presentations. Moreover, the biology of each distinct subtype is likely to differ. Although little systematic information has been collected regarding differences among various subtypes, we will later present some of our data regarding possible differences between two principal subgroups, melancholic and atypical depression.

Melancholic depression is a state of pathological hyperarousal and anxiety, most notably, about the self in the form of feelings of worthlessness and hopelessness about the prospects of a deficient self for future satisfaction in relationships or work. Thus, the term depression does not adequately capture this illness. Melancholic depression prevents experience of pleasure in what one has achieved or become, pleasures in current everyday life, and pleasures of a hopeful anticipation of a good future [8]. Melancholic depression can thus be seen as a state of anxiety and anguish that has infiltrated the entire cycle of life [8]. It is as if patients with melancholic depression were dominated almost exclusively by a preferential access to negatively charged emotional memories without adequate recall of explicit content. Prevailing data suggest that it is patients with melancholia who manifest evidence of an activated stress system, with alterations in a multiplicity of systemic processes affecting inflammation and metabolism, as well as multiple physiological signs of hyperarousal: hypercortisolism, suppression of the reproductive and growth hormone axes, insomnia (most often early morning awakening), loss of appetite, and loss of interest in sexual activity. Another feature of melancholia is a diurnal variation in the severity of depressed mood, which is most severe early in the morning (reviewed in [8]).

Although both atypical and melancholic depressions are associated with dysphoria and anhedonia, atypical depression seems the antithesis of melancholia. Atypical depression is associated with a disturbing sense of disconnectedness and emptiness. In contrast to patients with melancholic depression, who seem to have ready access to negatively charged memories, patients with atypical depression often seem walled off from themselves. They may complain of a cognitive and mental weariness and avoid others, often with the sense that contact would be too demanding and poorly received. Neurovegetative symptoms in atypical depression are the reverse of those in melancholia and consist of lethargy, fatigue, excessive sleepiness, increased food intake, weight gain, and depressed affect that worsens as the day progresses [22].

Only 25–30% of patients with major depression present with pure melancholic features while another 15–30% present with consistent atypical features [23]. Recent data from identical twin and family studies indicate that melancholic and atypical features are each heritable entities [24]. However, only a few studies of depression have stratified patients on the basis of the melancholic and atypical subtype.

In addition to melancholic and atypical major depression, other clinically relevant specifiers include psychotic depression, chronic depression, and severe depression, each of which may be associated with melancholic or atypical features. In addition, dysthymic disorders represent a chronic low grade but impairing depressive subtype that puts patients at risk for difficult to treat depressions. More than 75% of patients with dysthymia will go on to develop a major depression. In some, the dysthymic features will remain, imposing a burden termed double depression. Depressive disorders can coexist with other common axis 1 psychiatric disorders, complicating treatment issues. Depression with significant associated anxiety is often quite resistant to antidepressant regimens [25].

## 3. The Stress Response: Anatomic and Functional Template for the Pathophysiology of Major Depressive Disorder

### 3.1. Behavioral and Cognitive Components

The acute response to danger consists of a relatively stereotyped series of physiological and behavioral programs that promote survival during threatening situations. Physiological changes include increases in heart rate and blood pressure, shifts in blood flow to the brain and to the stressed body site, and breakdown of tissue in the mobilization of fuel. In addition, there is an inhibition of a repertoire of neurovegetative functions whose execution would be likely to diminish the likelihood of surviving a life-threatening situation (e.g., feeding, sleep, sexual behavior, and the endocrine programs for growth and reproduction) (reviewed in [4, 7–9]).

Fear-related behaviors predominate during stressful situations and are crucial for survival during emergencies. For this reason, an extensive circuitry for generating and modulating fear has evolved [21]. Depending on the context and constitutional factors (e.g., gender, stress system set point), fear leads to either defensive behavior that protects from harm or stimulates a struggle for survival. Speed and simplicity are essential, leading to a rapid deployment of simple, well-rehearsed behavioral, and cognitive responses. At the same time, there is an inhibition of more complex, novel, or untested responses that require considerable time to assemble [26].

Consistency is also essential for surviving stressful situations and is most apparent in the inhibition of mood shifts from one state to another. Thus, affect is often confined to a distressed, fearful mode. As noted, cognitive and behavioral repertoires are also relatively stereotyped during stressful situations. During the acute crisis, the mesolimbic dopaminergic reward system is stimulated to help maintain morale. During chronic stress or depression, the reward system is downregulated by stress mediators, resulting in relative anhedonia (reviewed in [5]).

### 3.2. The Corticotropin Releasing Hormone (CRH) and Locus Ceruleus-Norepinephrine (LC-NE) Systems during Stress

The CRH system contributes to the transduction of many of the components of the stress response. The intracerebroventricular infusion of CRH to the rat sets into motion intense arousal, multiple fear-related behaviors (e.g., inhibition of exploration, anxiety), and inhibition of neurovegetative functions that would be counterproductive during a threatening situation (e.g., sleep, feeding, and reproductive activity) (reviewed in [5]).

Two main components of the CRH system are the amygdala and hypothalamic CRH systems. The amygdala CRH system is largely responsible for the activation of anxiety and fear related behaviors, and CRH antagonists given ICV or systemically block fear conditioning. The amygdala CRH system also contributes to the activation of the hypothalamic CRH system and the LC-NE system.

The hypothalamic CRH system is responsible for the regulation of the hypothalamic-pituitary-adrenal axis and contributes to the activation of the LC-NE system. The hypothalamic CRH systems consist of two descending pathways. The first is humeral, in which CRH is released into the hypophyseal portal system to activate pituitary release of ACTH. A second pathway descends to the brainstem to areas such as the LC-NE system (reviewed in [5]).

There is also a peripheral CRH system that consists of CRH released from sympathetic nerve terminals to locally activate components of the innate immune system such as resident mast cells. CRH is among the most potent activators of mast cell degranulation. Administration of CRH antagonists to experimental animals attenuates experimentally induced autoimmune disorders such as adjuvant induced arthritis and myelin basic protein-induced encephalitis.

The LC-NE system is a general alarm system for the brain and increases the signal-to-noise ratio in key areas that regulate the stress response. It has many effects similar to those of CRH, such as the intense arousal, inhibition of neurovegetative functions, and reciprocally activates the amygdala and hypothalamic CRH systems. NE inhibits key functions of the prefrontal cortex and enhances the encoding and resistance to extinction of negatively charged emotional memories.

### 3.3. Insulin Resistance Is a Component of the Acute Stress Response

Acute insulin resistance is also an inherent component of the stress response. Stress mediators such as cortisol and cytokines lead to insulin resistance. In the context of insulin resistance, insulin dependent cells, such as muscle and fat, have less glucose transferred across their cell membranes per given amount of circulating insulin, so that plasma glucose levels rise. This is a bonus to the critical cells that do not depend upon insulin for glucose transport. These include the brain, the immune system, and the breast. Thus, during various stressors such as emotional stress or infection, the brain and immune systems have access to increased glucose needed to initiate and sustain increased activity of insulin receptor-independent structures. Similarly, during pregnancy and lactation, increased glucose is available for growth and increased metabolic activity.

### 3.4. The Acute Stress Response Is Associated with a Proinflammatory State

Multiple mechanisms set into motion an acute inflammatory response during exposure to stressors, thought to represent a premonitory readying of the immune system in case of acute injury incurred during a threatening situation. NE and other mediators activate the acute phase response, an important component of the innate immune response consisting of sixteen hepatic proteins that subserve multiple functions. C-reactive protein (CRP) and serum amyloid A (SAA) are among the two best-known proinflammatory mediators of the acute phase response [27], while fibrinogen is an important procoagulant compound. NE is among the stimuli of the acute phase response. Multiple inflammatory mediators stored in visceral fat are also released, including IL-6. Interestingly, the IL-6 response to stress is greater in obese individuals. Insulin is also a proinflammatory compound.

### 3.5. The Stress Response Is Also a Prothrombotic State

Along with insulin resistance and proinflammatory states, the stress response is a prothrombotic state as well. It is as if, during a stress response, a promontory procoagulant state is established as a stay to possible hemorrhage during threatening situations. As noted, fibrinogen is re leased as a part of the acute phase response.

Plasminogen activator inhibitor-1 (PAI-1), released by visceral fat, also participates in the acute stress response. As indicated by its name, PAI-1 inhibits plasminogen, a principal effector of the fibrinolytic system.

## 4. Proposed Circuitry of the Stress Response

### 4.1. The Prefrontal Cortex

The prefrontal cortex accounts for approximately one-third of human brain volume. In many respects, the prefrontal cortex exerts cognitive, behavioral, affective, and physiological responses that are the virtual antithesis of those set into motion during stress. The reciprocal of this relationship is also true, and the stress system inhibits the prefrontal cortex [28].

The subgenual medial prefrontal cortex is the area of the brain that determines whether the individual feels he or she is likely to experience punishment or reward. In addition, this area estimates the extent to which a realistic assessment has been rendered about an accomplishment or relationship. This area also provides cortical restraint upon the hypothalamic-pituitary-adrenal axis and the sympathetic nervous system [29, 30]. The medial prefrontal cortex is the only cortical brain structure that sends direct connections to the hypothalamus.

The prefrontal cortex is also actively involved in restraining the amygdala fear system and, among other tasks, promotes the extinction of aversively charged emotional memories [31]. Medial prefrontal cortex restraint of the amygdala fear system and its opposition to the process of conditioned fear are two of the important functions of the prefrontal cortex and very important in opposition to depressive illness. In many ways, areas of the prefrontal cortex transduce many key cognitive and emotional processes that transpire best in the context of relative freedom from an acutely threatening situation.

The prefrontal cortex arbitrates the bias towards two cognitive domains and, as noted, determines the extent to which complex, newly synthesized, novel programs predominate versus the extent to which these are suspended in favor of relatively reflexive responses that generically offer protection against threatening stimuli. An activated prefrontal cortex promotes complex cognitive programs and inhibits those that are more reflexive. The prefrontal cortex is also essential for the appropriate shift of one affective state to another [28]. A shifting affect is not adaptive during a dangerous situation, during which affect should be clamped in a fearful mode rather than having the capacity to range broadly to stimuli that may be distracting.

### 4.2. The Amygdala Fear System

Because fear is essential for surviving serious threats, the stress system must be capable of producing the experience of being afraid. The amygdala is a key structure that transforms experiences into feeling [21]. To accomplish this task, the amygdala provides working memory with further information about whether something is good or bad and activates disparate arousal centers to maintain focus upon the current danger. The amygdala evolved relatively early compared to higher cortical centers.

The amygdala is responsible for acquiring and storing classic fear conditioned responses that can be immediately mobilized even though they remain outside of conscious awareness. Because the amygdala cannot store complex, explicit aversively charged emotional memories, it relays them to areas such as the hippocampus and striatum for retrieval during subsequent emergencies. The amygdala is a key structure for receiving and either encoding or transmitting neural information regarding aversively charged emotional memories [26, 31]. Emotional memories are very well remembered. They are stored in sites distinct from those that store and transmit knowledge regarding everyday functioning in a material world such as spatial memory [32] and other forms of nonemotionally laden memories [33].

The distinct loci for explicit and emotional memory [33] are demonstrated by the phenomenon in which individuals with damage to explicit, fact-laden memories but intact sites for emotional memories respond to someone who had frightened them by crying out, without recognizing the person or the context in which they were frightened. They say, instead, that they have no idea why they became frightened, but only that they are frightened without apparent cause. This emergence of an unconscious emotional memory without apparent content occurs frequently in everyday life and in patients under treatment for depression.

Thus, in response to a situation resembling that explicit event or place in which the upsetting experience occurred, an emotional response can occur in the absence of a conscious awareness of the context in which it was acquired [20, 21, 26, 28, 34]. As noted later, the imprinting of emotional memory is greatly strengthened by the classic hormones secreted during stress. This makes sense because it is adaptive to increasing the strength of an emotional memory's encoding so that it can emerge unconsciously to signal an individual that danger is imminent, including not only physical danger, but the danger of abandonment, loss, or humiliation.

### 4.3. Hippocampus

The hippocampus is well known for its effects on various components of memory of everyday events, words, faces, and stories, including the stories of individual lives. Recent data suggest that the hippocampus is also involved in the encoding of aversively charged memories, on the one hand, and the extinction of aversively charged memory, on the other. It is also well known that the hippocampus is crucial to the remembering of place, and other details that may have been associated with prior threatening situations [34]. These memories emerge virtually reflexively and unconsciously with exposure to other potential threatening stimuli (Cahill, McGraw, 1998) [35]. These can be recalled reflexively when exploring a context similar to one where prior threats occurred. The hippocampus also shares the role with the mPFC of retraining the stress response when it is no longer necessary, including HPA axis responses [29]. During controllable stress, nerve terminals sprout and new neurons are born and the acute stress response subsides [36]. In uncontrollable stress, the nerve terminals do not sprout, but rather shrink. In addition, there is a marked diminution in the birth of new neurons. These abnormalities are reversed by antidepressants, in association with normalization of the stress response and behavioral inhibition [37].

### 4.4. Nucleus Accumbens

The nucleus accumbens are one of the main areas of the brain that responds to dopamine stimulation, conferred by neurons that lie predominantly in the brain stem. The nucleus accumbens are responsible for the experience of pleasure derived from multiple sources including sex, eating, and the enjoyment of ideas and other people [38]. Its structure and function are significantly altered in depressive disorders [39].

## 5. Pathophysiology of Major Depression as a Dysregulation of the Stress Response Depression

### 5.1. Activation of the CRH System

Activation of the CRH system is a hallmark of major depression with melancholic features associated with hypercortisolism. Thus, in contrast to the pituitary-mediated hypercortisolism in Cushing's disease, hypercortisolism in major depression is of a central origin [40]. The hypercortisolism of melancholia is most distinct in the early morning hours, when cortisol levels are at their nadir. This defect is best demonstrated by CRH and CRH/dex testing. Gold et al. also showed normal CSF CRH levels taken hourly via a lumbar drain hourly for 24 hours. Although apparently contradictory, normal CSF CRH levels taken hourly for twenty-four hours in melancholic depressed patients were inappropriately in the normal range in the context of pronounced hypercortisolism, which should have suppressed hypothalamic CRH neurons that release CRH into the CSF [41]. Later, Gold et al. found that cortisol, though suppressing hypothalamic CRH, actually activated the amygdala CRH system. Thus, CSF CRH is modulated by glucocorticoids in antithetical ways.

### 5.2. Activation of the Locus Ceruleus-Norepinephrine System

The original catecholamine hypothesis of major depression stated that depression resulted from a deficiency of NE at critical synapses in the CNS. This hypothesis was based on the assumptions that pharmacological depletion of NE by reserpine apparently induced major depression, while apparent pharmacological augmentation of noradrenergic activity by MAO inhibitors and NE uptake inhibitors (tricyclic antidepressants) exerted antidepressant effects. By positing that depression could be caused by a deficiency of NE, the catecholamine hypothesis served as a major impetus for the emergence of modern biological psychiatry. However, as research became more sophisticated, it became clear that early life stress profoundly increases the activity of all stress components throughout the life of the individual (vide infra). It is now unequivocal that environmental factors are critical to the development of noradrenergic abnormalities in depression.

Although some forms of depression may be characterized by a deficiency of NE in the CNS, melancholic depression is associated with increased noradrenergic function in the CNS [41]. Decreased flexibility of mood and cognition, hyperarousal, increased anxiety, decreased feeding, and insomnia could all be driven by norepinephrine excess. This is compatible with the effects of NE in the CNS to inhibit the medial prefrontal cortex, activate the amygdala, and activate the CRH-cortisol system. We have shown that the secretion of cerebrospinal fluid NE is elevated around the clock in patients with melancholic depression [41]. Reference [4] also showed that the increased around-the-clock CSF NE secretion was paralleled by increased around-the-clock plasma NE levels as well. The levels rise throughout the night, during sleep, and peak in the morning at the time when patients with melancholic depression experience the most intense depression. This is also the time of maximal vulnerability to myocardial infarction and may contribute to the increased mortality and premature heart disease in patients with major depression, to be covered below. Thus, the CNS mediators that contribute to depression also contribute to its long-term medical consequences, establishing major depression as a serious systemic disease.

Recent data further support the presence of an activated locus ceruleus-norepinephrine system in major depression. The SSRIs, tricyclic antidepressants, and MAO inhibitors all decrease the firing rate of the locus ceruleus in freely moving rats. We found that imipramine [42], fluoxetine [43], and an MAO inhibitor [43] downregulated the expression of the rate-limiting enzyme in catecholamine synthesis in the locus ceruleus.

The serotonergic system has received some of the widest coverage among extracellular mediators. This is undoubtedly true because of the wide utilization of SSRI's in the treatment of depressive syndromes. The weight of available data suggests that SSRIs reduce the sensitivity of presynaptic 5HT2 receptors, thus augmenting the relapse of serotonin into the synapse. The mechanism is more complex, but given the multiplicity of serotonin receptor subtypes and its pleiotropic interactions with other neural mediators, a further discussion of this subject is beyond the focus of this review. Dopamine and glutamate are also clearly implicated in major depression. Dopamine is a principal mediator of pleasure and reward and its activity may be diminished in patients with major depression. Glutamate is also implicated and to date evidence suggests that NMDA, MGluR-l, and mGluR-5 receptor antagonists all ameliorate depression while pharmacologic stimulation of the AMPA receptor promotes recovery from depression.

There have been few systematic studies examining differential pathophysiology of melancholic and atypical depression. The weight of available data suggests that the pathologic activation of the stress system in patients with major depression, with activation of the CRH and LC-NE systems, occurs predominantly in patients with melancholic depression, though this point is far from being definitively established.

As noted, atypical depression seems the antithesis of the symptom complex of melancholia. We first hypothesized that the lethargy, fatigue, and hypersomnia of atypical depression were associated with a pathological reduction of stress system activity [7–9]. This possibility was supported in the previously cited data in experimental animals showing that specific lesions in a specific part of the prefrontal cortex resulted in pathological suppression of the core stress system. Our data based on studies of different components of the HPA axis in fatigue states such as chronic fatigue syndrome and seasonal affective disorder suggests that the HPA axis is hypoactive in states that resemble atypical depression [44, 45].

The etiology or antecedents of the possible suppression of the stress system have not been elucidated. Rene Spitz and others made invaluable observations regarding developmental abnormalities that befell infants placed in understaffed orphanages with a dearth of human contact shortly after birth. For the first five or six months, the infants cried bitterly for hours until attended. Subsequently, they withdrew and their crying ceased altogether, even if they were left alone or had gone without eating for many hours. In addition, they lost interest in the environment around them. It was as if the trauma of their early deprivation had led to a shutdown of their affective existence to protect them from unendurable pain. Subsequent studies in nonhuman primates who were abandoned or abused reveal a similar behavioral withdrawal in association with hypoactivity of the HPA axis [46].

## 6. Focus Extended to Inflammatory, Metabolic, and Oxidative Stressors at the Cellular Level and Processes That Promote Homeostasis Such as Neuroplasticity and Neurogenesis

Recent data have widened the field considerably to consider the presence of epigenetic factors as well as of inflammatory, oxidative, and metabolic stressors at the cellular level. A prolific literature documents a clear proinflammatory state in patients with depressive illness, a dysregulation with many implications not only for inflammation but neuronal energy metabolism as well.

The inflammation in depressive illness, at first glance, appears to be a mild smoldering activation of the innate immune system. These clinical studies are supported by findings in experimental animals, which show that sufficiently severe psychological and physical stressors can produce depression-like and proinflammatory states that are both reversed by antidepressant treatments.

In response to stressors, adaptive neuroplastic changes as well as increases in neurogenesis in the hippocampus emerge to promote the maintenance of homeostasis. In animal models of depression precipitated by severe stressors, there is an inhibition of neuroplasticity and neurogenesis that is reversed effectively by antidepressant treatment [47, 48].

The etiology of the defects in these adaptive mechanisms in response to stressors is unknown. A recent study found a significant increase in MAP kinase phosphatase-1 (MKP-1) in hippocampi taken from postmortem brains of patients who had committed suicide. In experimental studies, rats exposed to depression-inducing conditions show significant increases in hippocampal MKP-1 gene expression, which are ameliorated by antidepressants. MPK-1 is a negative regulator of the map kinase cascade, a major signaling pathway involved in neuronal plasticity, function, and survival [49].

Though no causal relationship has yet been established between deficits in the capacity to respond to stress with adaptive neuroplastic and neurogenetic responses, patients with depressive illness have impairments in cellular number and size in key areas of the brain that are likely to play important roles in depressive illness. These are detailed in the following section.

## 7. Neuroimaging Studies of Stress System Components in Major Depression

### 7.1. The Prefrontal Cortex in Major Depression

A key finding that has been well replicated is that of a significant loss of volume in the left subgenual prefrontal cortex, an area that that plays a key role in the estimation of the likelihood of punishment or reward. Thus, this deficit leaves the individual vulnerable to a significant increase in the expectation of harm. This area is closely connected to the amygdala and inhibits the amygdala fear system, and it confers the impact of the cerebral cortex in the inhibition of the HPA axis and sympathetic nervous system [30]. These patients were predominantly individuals with melancholic depression (W. Drevets, personal communication). Scanning and examination of postmortem brain samples taken from patients who had committed suicide revealed a forty percent decrease in the volume of the left subgenual cortex. It is of interest that the lateralization found in patients with depression is compatible with data in the rat showing that lesioning of the left infralimbic cortex causes activation of the HPA axis and of sympathetic function, while lesioning of the right produced a decrement in the activity of these systems. These data indicate that the left infralimbic region inhibits the right. We therefore postulate that the left-sided defect in melancholic depressed patients leads to hyperactivity of both the amygdala and core stress system components. In patients with atypical depression, however, the left could be hyperactive or hypertrophied, leading to excessive restraint of the right and hypoactivity of core stress system components.

A series of studies in patients with major depression have reported significant decreases in activation of the dorsolateral prefrontal cortex and significant increases in ventral prefrontal and paralimbic structures. Higher depression ratings correlated negatively with the activity of left dorsolateral prefrontal cortex, while anxiety levels were positively correlated with paralimbic system activity. Successful treatment of depression was associated with inhibition of overactive paralimbic regions and normalization of hypoactive dorsolateral prefrontal cortex sites. Thus, major depression seems associated with hypoactivity of cortical structures and a corresponding hyperactivity of paralimbic/subcortical loci.

### 7.2. The Amygdala in Major Depression

Patients with major depression show increased cerebral blood flow and metabolism in the amygdala. Activation in the left amygdala persisted after recovery from depression. During depression, amygdala activation correlated positively with depression severity and baseline plasma cortisol levels. The latter finding is of interest in the light of the fact that the amygdala activates the HPA axis. Glucocorticoids, in turn, accentuate the amygdala CRH system [50]. A recent study found that neural activity in several 5-HT-related brain areas, for example, dorsal raphe, habenula, septal region, amygdala, and orbitofrontal cortex, covaried significantly with plasma levels of tryptophan and ratings of depressed mood. Antidepressant treated patients who relapsed upon tryptophan depletion had higher baseline amygdala metabolism that similar subjects who do not relapse.

## 8. Depression as a Complex Disorder of Adaptation

2500 years ago, Hippocrates advanced the principle that we are continually beset by disturbing forces that threaten to upset the interior balance upon which our lives depend. Fortunately, he noted, there are counteracting forces that oppose the disturbing forces and work to maintain or restore homeostasis. Galen called these Vis medicatrix naturae the healing forces of nature. For the purpose of this overview, we will call the disturbing forces stressors, the balance, homeostasis, and the counteracting forces and adaptive responses. According to this schema, we can define stress as an ongoing or perceived state of threatened homeostasis.

We have subsequently learned that the adaptive forces themselves can become disturbing forces or stressors that threaten homeostasis. Autoimmune disorders are perhaps the most thoroughly elucidated. We need our immune system to survive a myriad of disturbing forces, and it is among our most critical of our adaptive responses. However, the survival value of the immune response can be circumvented by a dysregulation of the immune adaptive response that becomes either hyperactive or inappropriately directed at the self. Major depression is also a disorder of adaptation and can be conceived, in part, as a dysregulation of the stress response. For humans, this dysregulation involves a perception of the world that provokes fear and dread of the future and a perception of self that is full of anxiety and dissatisfaction. Thus, depression can also be considered a disorder of the self-imposed by a disorder of an adaptive response.

Disorders of the immune system occur in the context of multiple predisposing genes and a predisposing environment. For autoimmune disease, the predisposing environment is one that contains a specific inciting antigen or repertoire of antigens that provokes an excessive or self-directed immune response. For depression, the predisposing factors include the burdens of internal and external conflicts, and the sum and intensity of stored aversively charged emotional memories of abandonment, unkind treatment, or abuse.

It is well documented that disturbing events at critical periods early in life are particularly well encoded in emotional memory and alter the physiology and phenomenology of the stress response and of self-perception for the rest of the individual's life. Indeed, in experimental animals, early stress such as relatively short maternal separation turns up the intensity of the stress response for the remainder of the organism's life. Conversely, early gentle handling and lack of interference with the maternal-offspring bond are associated with a relatively restrained stress response throughout life [51, 52].

The intersection of stressful and disturbing life experiences encoded in emotional memory, as well as genetic vulnerability, [53] provides the context for understanding how psychotherapeutic intervention can influence a disorder with biological vulnerability that is responsive to somatic treatments.

## 9. Brief Clinical Overview of the Treatment of Major Depression with Emphasis on Partial Response or Treatment Resistance

A broad coverage of treatment strategies in the treatment of major depression is beyond the scope of this paper. In response to first line treatments such as SSRIs, SNRIs, or bupropion, only approximately a third of patients achieve a full remission. Another 30% remitted after a second form of treatment. The remaining third achieved only a partial response or had no response at all. The second line treatment of patients with major depression has been the subject of a great deal of attention, and I will briefly cover here an overview of current conclusions.

Most feel after failing to achieve remission in the first round of treatment, a second agent should be added, and the first kept on board unless there was no response at all or intolerable side effects. This prevents a loss of any of the efficacy of the first compound and a withdrawal that can be difficult as well as leading to delay of 2nd round treatment. Adding a given agent presumes that the initial therapy was of sufficient duration (4–6 weeks) and of adequate dosage.

The most effective strategy for augmentation was the addition of an antipsychotic, either Abilify or Zyprexa. Abilify is preferred because it has less of propensity to cause significant weight gain. Slightly less effective was the addition of mirtazapine and omega 3 fatty acids, and a slight notch below was the addition of bupropion with the addition of either mirtazapine or omega-3 fatty acids. Modafinil lithium and T3 were slightly less effective but were superior to placebo. These were followed by mecamylamine, an anticholinergic agent, and desipramine. Pindolol, buspirone, and lamotrigine were less effective but did promote remission superior to that of placebo.

*Hazards of Newer Antidepressants*. Although being safer than tricyclic antidepressants and monoamine oxidase inhibitors, the newer antidepressants may be associated with certain medically serious adverse effects, including cardiovascular adverse effects such as hypertension, seizures, abnormal bleeding, agranulocytosis, and hyponatremia [54]. Their main advantage is that they are generally better tolerated and significantly safer in an overdose than tricyclic antidepressants and MAO inhibitors. However, in cases of severe melancholic depression, the tricyclic antidepressants are more effective, while the MAO inhibitors may be more effective in severe atypical depression. On the other hand, tricyclic antidepressants are notably ineffective in atypical depression [55].

*Cardiovascular Effects.* The SSRIs are generally safe for treating patients with hypertension or cardiac disease. In patients with significant cardiac disease, patients had a significantly lower incidence of cardiac side effects (2%) versus nortriptyline (18%). The predominant side effect of the tricyclic was increased heart rate, although conduction abnormalities were also noted. In contrast to tricyclics and MAO inhibitors, SSRIs produced no significant increase in QT intervals. Thus, SSRIs are safe even in those who have had acute MIs.

SNRIs are also safer than the older antidepressants. However, above 225 mg of venlafaxine, 10% of older male patients will experience the new onset of hypertension. Venlafaxine generally did not worsen hypertension in those already under effective treatment. Generally, venlafaxine is associated with a small increase in heart rate and a dose-dependent increase in blood pressure. Venlafaxine has not been reported to have adverse effects on cardiac contractility. For single drug overdoses, the death rate per million prescriptions for venlafaxine was higher than that for SSRIs (13.2 versus 0.7). In general, venlafaxine is associated with a higher risk of cardiac side effects than duloxetine or milnacipran. Venlafaxine is metabolized mainly by CYP2D6, as is bupropion, so that the doses of these medications should be modified when given together. At ordinary doses, duloxetine has only a very modest effect on heart rate and blood pressure and no demonstrable effect on the QT interval. At high doses, both systolic and diastolic pressure will increase a mean of 10 mm/hg systolic and 5 diastolic [55].

Bupropion is not associated with significant cardiac side effects when prescribed to healthy people in appropriate doses. In depressed patients with preexisting heart disease, it may be associated with a rise in blood pressure and orthostatic hypotension, although it does not cause conduction delays. Bupropion overdose is associated with cardiovascular toxicity including tachycardia and conduction delays [55].

Mirtazapine generally does not have significant effects on heart rate or blood pressure. However, four cases of torsades de pointes variation of ventricular tachycardia have been noted. In at least three of the cases, other drugs were implicated; all were recovered. Mirtazapine has proven safe in patients with recent myocardial infarction. Of 23 patients with documented overdoses, three developed tachycardias, one bradycardia, and one had moderate but not life threatening hypotension. There were no conduction delays. Apart from direct cardiovascular effects, mirtazapine can produce weight gain, resulting in metabolic syndrome and cardiac disease as a result, in part, of endocrine complications (e.g., insulin resistance) and dyslipidemia. 

*Seizures.* Seizure incidence in patients on bupropion is heavily dose dependent, with rates exceeding 1% at doses greater than 450 mg. The corresponding rate in SSRI-treated patients is at least ten times lower. At 300 mg, the rate is about 0.1%, a rate similar to other newer antidepressants. These rates are significantly lower than for tricyclics, clozapine, and phenothiazines. The risk of bupropion-induced seizures is greater in those predisposed to seizures. The risk of seizures on patients taking SNRIs is lower than that for tricyclics and not very different from the first incidence of seizure in the general population. 

*Bleeding*. Serotonergic antidepressants are associated with bleeding, of GI origin in particular. Serotonin plays an important role in platelet aggregation, and blockade of the 5HT transporter inhibits platelet aggregation and may lead to bleeding. The most potent 5HT uptake blockers, fluoxetine, sertraline, or paroxetine are the most likely offenders. The risk is modest (relative risk of GI hemorrhage 3.6 compared to general population) but increases significantly on patients taking NSAIDs or aspirin. SSRIs have also been reported to increase vasospasm after subarachnoid hemorrhage and adversely influence outcome. It should be noted that SSRIs affect platelet aggregation but do not affect coagulation tests such as the prothrombin time [55]. 

*Hyponatremia*. Several antidepressants have been associated with hyponatremia due to the inappropriate secretion of vasopressin, the antidiuretic hormone. Although being unusual in younger patients, the risk rises markedly with the elderly, with other risk factors including low body weight, female sex, previous history of hyponatremia, salt wasting drugs (e.g., diuretic, NSAIDS), and a lower plasma Na level (<135 meq/L). Hyponatremia usually occurs within three weeks on treatment initiation and may be transient or persist. SSRIs, SNRIs, and bupropion have all been associated with hyponatremia. Sodium administration does not correct the problem. Carefully supervised fluid restriction as well as demeclocycline may help, but treatment must be supervised by an endocrinologist. 

*Agranulocytosis*. Defined as a decrease in neutrophil count below 500 cells/mm^3^, it has been primarily associated with tricyclics and tetracyclics, but the newer antidepressants can also lead to this life-threatening complication. Among the newer antidepressants, mirtazapine has been rarely associated with agranulocytosis. Most cases occurred in those with other risk factors for agranulocytosis, including patients with HIV, cancer, those on cancer chemotherapy, and drugs known to cause agranulocytosis, including carbamazepine and depakote [55]. 

*Mechanism of Action of Psychotropic Drugs*. The mechanisms of action of antidepressants are apparently all involved with or dependent upon the alteration of a number of neuromediators. This reflects either the fact that these drugs necessarily exert multiple actions in order to work, or after a key initial impact, a series of other related compounds are influenced in a quantifiable fashion. A detailed discussion of this topic is beyond the scope of this review. The effects of many psychotropic drugs intersect at one or more systems or mediators. In terms of stress mediators, we have shown that SSRIs, tricyclics, and MAO inhibitors given chronically downregulate the CRH and LC NE system. These agents also ameliorate the proinflammatory response to stress in experimental animals as well as stress-induced loss of adaptive neuroplasticity and neurogenesis. Thus, compounds which may play a key role in the pathophysiology of major depression and its phenotypic expression also responds to common antidepressant agents.

## 10. Summary

Melancholic and atypical depression can be conceptualized as dysregulations of the stress system response, and, hence, as disorders of adaptation. The dysregulation in stress system mediators in these disorders confers many of their clinical and biochemical manifestations. In melancholia, prefrontal cortex and amygdala dysregulation produce an increased expectation of harm, anxiety, and disinhibition of the HPA axis and LC-NE systems. This further complicates the hyperarousal and general alarm of this disorder. In addition, the disinhibition of stress mediators contributes to the systemic manifestations of major depression such as premature coronary artery disease and premature osteoporosis. Similarly, antidepressants effective in melancholia such as tricyclics, specific serotonin reuptake inhibitors, and MAO inhibitors downregulate principal effectors of the stress response such as the CRH and LC-NE systems and correct dysregulations of the subgenual prefrontal cortex and the amygdala to return them itto their nonstressed states.

Severe stress produces depression like pictures in experimental animals associated with disruption of counter-regulatory stress system responses such as adaptive neuroplasticity and neurogenesis. These deficits are also corrected by standard antidepressant treatment. Such findings may help in the elucidation of the pathophysiology of major depression and provide targets for new therapeutic interventions. The advent of studies of intracellular stress mediators and genomic medicine opens up entirely new avenues for research and discovery relating to major depression, now the fourth leading cause of disability worldwide. Studying genomic aspects of depressive illnesses is substantially complicated by the fact that depression is a polygenic disease, so that genetic contributions are likely to be the result of multiple gene, each having relatively small effects. Moreover, we have yet to identify an endophenotype of depressive illness based on biological alterations. Such an achievement would assist enormously in diagnosing patients with specific subtypes of depressive illness, decreasing the heterogeneity of patients who are now selected for biological investigations. The Connectome Project is in its relative infancy and will reveal connections among CNS structures that we are currently unable to identify. We are currently on the threshold of learning more about depression in the next few years than we have over the past seventy years. The refinement of current technologies and the emergence of new ones will have a profound effect on efforts to uncover basic mechanisms underlying depressive illness.
